# Cardiovascular Disease Mortality and Serum Carotenoid Levels: a Japanese Population-based Follow-up Study

**DOI:** 10.2188/jea.16.154

**Published:** 2006-07-13

**Authors:** Yoshinori Ito, Mio Kurata, Koji Suzuki, Nobuyuki Hamajima, Hitoshi Hishida, Kunio Aoki

**Affiliations:** 1 Department of Preventive Medicine/Biostatistics and Medical Decision Making, Nagoya University Graduate School of Medicine; 2 Department of Public Health, Fujita Health University School of Health Sciences; 3 Department of Internal Medicine, Fujita Health University School of Medicine

**Keywords:** Follow-Up studies, Cardiovascular Diseases, lycopene, Carotenoids, Japan

## Abstract

**BACKGROUND:**

Some observational epidemiologic studies suggest that dietary and serum carotenoids are associated with reduced cardiovascular disease mortality.

**METHODS:**

Three thousand and sixty-one subjects (1,190 males and 1,871 females), aged 39 to 80 years, were recruited from residents of Hokkaido, Japan who had attended comprehensive health check-up programs from 1988 through 1995. Serum levels of *α*-carotene, *β*-carotene, and lycopene were separately determined by high-performance liquid chromatography. Serum levels of total carotene consisted of the sum of *α*-carotene, *β*-carotene, and lycopene levels. Each serum level of *α*-carotene, *β*-carotene, lycopene, total carotene, triglyceride, and alanine transaminase (ALT) activity was transformed logarithmically. The hazard ratios of serum *α*- and *β*-carotenes, lycopene, and total carotene values were estimated by the Cox proportional hazard model after adjusting for sex, age, and other potential confounding factors.

**RESULTS:**

During the 11.9-year follow-up period, 80 deaths (49 males and 31 females) from cardiovascular disease, 40 deaths from heart disease, and 37 deaths from stroke were identified among the cohort subjects. High serum values of carotenoids such as *α*- and *β*-carotenes, and lycopene were found to be significantly associated with low hazard ratios for cardiovascular disease mortality. However, a significant inverse association between high serum lycopene value and the risk for stroke mortality was not always observed.

**CONCLUSIONS:**

High serum levels of total carotene, comprising *α*- and *β*-carotenes and lycopene, may reduce the risk for cardiovascular disease mortality among the Japanese population.

Many observational epidemiologic studies have shown that a high dietary intake of fruit and vegetables rich in carotenoids, such as *α*- and *β*-carotenes, is associated with reduced cardiovascular disease mortality, including coronary heart disease and stroke.^1^^-^^6^ Many epidemiologic studies have shown that high dietary intakes or high serum levels of carotenoids are also associated with a low risk for coronary heart disease^7^^-^^13^ and stroke^14^^,^^15^ mortality. In contrast, a significant association between high dietary intakes or high serum levels of lycopene and a low risk of coronary heart disease or stroke were not always reported among Europeans.^16^^-^^18^ However, no report has examined whether high serum levels of *α*-carotene and *β*-carotene are inversely related to a cardiovascular disease mortality risk in a Japanese population-based follow-up study. It has been suggested that cardiovascular disease mortality risk is reduced by carotenoids due to their role in protecting against oxidative stress.^19^^-^^22^

In the present research, we studies whether high serum levels of *α*-carotene and *β*-carotene as well as lycopene are associated with a reduced risk of death from cardiovascular disease, including heart disease and stroke, by doing a follow-up study among Japanese subjects.

## METHODS

### Subjects and Comprehensive Health Check-up Programs

The study subjects were recruited from residents of a rural area of Hokkaido, Japan, who attended a comprehensive health check-up programs^23^^,^^24^ from 1988 through 1995. Among the 3,182 inhabitants who first attended those programs, 3,061 participants (1,190 males and 1,871 females) were enrolled after excluding subjects under medical treatment and/or those using medicines for heart disease and/or stroke (50 males and 71 females). Although the comprehensive health check-up programs were offered to individuals 40 years of age and over, individuals 39 years old were accepted if they turned 40 during that year. The subjects were not randomly selected. Initial subjects were those who attended the programs and were 40, 50, and 60 years of age. Once those subjects were enrolled, any residents 40 years or over were accepted until 1,000 subjects were seen each year.

Information used in this study was obtained from a questionnaire, and the serum samples collected at the time of the comprehensive health check-up programs. All subjects gave their written informed consent for using their information and sera in an epidemiologic study of life-style-related disease prevention.^25^ A questionnaire regarding health status, personal medical-treatment history, and daily lifestyles was distributed to the participants a few days before the health check-up programs, and the answers were checked and revised by trained health nurses during the programs.^23^^-^^25^ The responses on smoking status and alcohol consumption were obtained by health nurses during individual interviews using a three-level questionnaire: (1) Current smoker (cigarettes smoked daily); (2) Former smoker (quit smoking); and (3) Never smoker, and (1) Regular drinker (alcohol consumed daily); (2) Irregular drinker (alcohol consumed occasionally); and (3) Non drinker (alcohol never consumed).

### Determination of Serum Levels

Fasting serum samples were taken at the time of the comprehensive health check-up programs, and then serum levels of *α*-carotene, *β*-carotene, and lycopene were separately determined using high performance liquid chromatography, as previously reported.^26^ Serum levels of total carotene consisted of the sum of the *α*-carotene, *β*-carotene, and lycopene levels. Serum levels of total cholesterol, triglyceride, alanine transaminase (ALT) activity, and other biochemical tests were determined using an auto-analyzer (SMAC, Technicon Co., Ltd., or TBA-80M, Toshiba Co., Ltd).

### Follow-up of the Cohort

The cohort was followed until December 2003 using mortality records obtained with the permission of the Agency of General Affairs and the Ministry of Health and Welfare. Death certificates were examined to check the direct and indirect causes of death, which were classified using the International Classification of Diseases (ICD) 9th from 1989 through 1994 and ICD 10th from 1995 through 2003.

The number of residents who moved away, excluding the young age group, was small (males, 9%; females, 12.3%),^25^ and the number of deaths was reliable. We calculated the sample death rates using person-years.

### Statistical Analysis

Cardiovascular disease mortality rates were calculated as the number of deaths per person-year for each serum *α*-carotene, *β*-carotene, lycopene, and total carotene level. Each serum value of *α*-carotene, *β*-carotene, lycopene, total carotene, triglyceride, and ALT activity was transformed logarithmically. The hazard ratios per each logarithmically transformed value of serum *α*- and *β*-carotenes, lycopene, and total carotene levels (*μ*mol/L) for the mortality from cardiovascular disease were estimated by the Cox proportional hazard models (JMP^®^ ver 4.0) as follows: Model 1, adjusted for sex, age, and smoking status; Model 2, adjusted for sex, age, smoking status, alcohol consumption, body mass index (BMI= weigh kg/ height m^2^), systolic blood pressure, serum total cholesterol level, and serum logarithmically transformed values of triglyceride and ALT activity. An analysis of the mean differences for serum values of carotenoids, such as *α*- and *β*-carotenes and lycopene, and total carotene between those who died and the survivors was conducted using analysis of variance (ANOVA) after adjusting for age. Statistical significance was set at p <0.05.

## RESULTS

### Comparison of Baseline Serum Carotenoid Levels for Subjects Who Survived and Those Who Did Not

Table 1 shows age and sex distributions, smoking status, and alcohol consumption of the subjects at baseline and the causes of deaths. About 30% were 39-69 years old; subjects 70 to 80 years of age comprised less than 10% of the cohort. The proportions of current smokers and regular drinkers were 52.4% and 76.4% for males and 11.1% and 29.1% for females, respectively. Overall, 16.6% of study subjects had an obvious history of medical treatment for hypertension, while 2.9% had clearly undergone medical treatment for diabetes. A high BMI (above 25 kg/m^2^) was seen in 36.1%, while 6.4% had high ALT activity (over 40 U/L), 40.2% showed high serum cholesterol levels (above 220 mg/dL), and 23.1% had high serum triglyceride levels (above 150 mg/dL). During the 8- to 15-year follow-up period (mean: 11.9 years), 301 deaths (181 males and 120 females) occurred from all causes, 139 from all types of cancer, 59 from other diseases, and 23 from external causes. The 80 deaths (49 males and 31 females) were attributed to cardiovascular disease, comprising 40 from heart disease, 37 from stroke, and 3 from other causes of cardiovascular disease.

**Table 1.** Baseline characteristics of study subjects (Number of cases).

	Males	Females	Both sexes
Total	1,190 (100)	1,871 (100)	3,061 (100)

Age (years)			
39-49	437 (36.7)	713 (38.1)	1,150 (37.6)
50-59	337 (28.3)	599 (32.0)	936 (30.6)
60-69	327 (27.5)	460 (24.6)	787 (25.7)
70-80	89 (9.5)	99 (5.3)	188 (61)

Smoking status			
Current smoker	623 (52.4)	207 (11.1)	830 (27.1)
Former smoker	291 (24.4)	74 (4.0)	365 (11.9)
Never smoker	276 (23.2)	1,590 (85.0)	1,866 (61.0)

Alcohol consumption			
Regular drinker	909 (76.4)	544 (544.1)	1453 (47.5)
Irregular drinker	53 (4.4)	37 (2.0)	90 (2.9)
Non-drinker	228 (19.2)	1,290 (68.9)	1,518 (49.6)

History of diseases			
Hypertension	172 (14.5)	329 (17.6)	508 (16.6)
Diabetes	50 (4.2)	40 (2.0)	90 (2.9)

Findings			
Body mass index 25.0+ kg/m^2^	436 (36.6)	669 (35.8)	1,105 (36.1)
Alanine transaminase 41+ U/L	117 (9.8)	80 (4.3)	197 (6.4.)
Total cholesterol 220+ mg/dL	422 (35.5)	809 (43.2)	1,231 (40.2)
Triglyceride 150+ mg/dL	354 (29.7)	353 (18.9)	707 (23.1)

Cause of death			
All causes	181 (100)	120 (100)	301 (100)
Cardiovascular disease	49 (27.1)	31 (25.3)	80 (26.6)
Heart disease	24 (13.2)	16 (13.3)	40 (13.3)
Stroke	24 (13.2)	13 (10.8)	37 (12.3)
Others	1 (0.1)	2 (1.7)	3 (1.0)
Cancer of all sites	83 (45.1)	56 (46.7)	139 (26.6)
External causes	12 (6.6)	11 (9.2)	23 (7.6)
Others	37 (20.4)	22 (18.3)	59 (19.6)

Percentages in parentheses

For both males and females, serum geometric mean values of *α*-carotene, *β*-carotene, lycopene, and total carotene tended to be lower for those who had died due to cardiovascular disease (including heart disease and stroke) than for those who survived (Table 2). The serum values of *β*-carotene in females were significantly lower for those who had died of cardiovascular disease (including stroke) than for those who survived. Alpha-carotene values for males and lycopene values for females were also significantly lower among those who had died of heart disease than among those who survived. In contrast, serum lycopene values for females tended to be slightly higher for those who had died due to stroke than for those who had remained alive.

**Table 2.** Comparison of baseline geometric means of serum carotenoid levels between those who died and survivors.

Serum carotenoids	Sex	Alive*	Cardiovascular death^†^	Heart disease death^†^	Stroke death^†^
*α*-carotene (*μ*mol/L)	Males	0.106	0.090 (0.076)	0.081 (0.034)	0.095 (0.391)
	Females	0.166	0.146 (0.190)	0.147 (0.370)	0.145 (0.371)

*β*-carotene (*μ*mol/L)	Males	0.425	0.388 (0.400)	0.413 (0.876)	0.356 (0.235)
	Females	0.969	0.759 (0.025)	0.864 (0.461)	0.679 (0.033)

Lycopene (*μ*mol/L)	Males	0.258	0.221 (0.142)	0.237 (0.607)	0.194 (0.056)
	Females	0.371	0.327 (0.304)	0.248 (0.017)	0.424 (0.539)

Total carotene (*μ*mol/L)	Males	0.848	0.750 (0.168)	0.789 (0.591)	0.685 (0.091)
	Females	1.587	1.321 (0.057)	1.343 (0.214)	1.296 (0.172)

n	Males	1140	49	24	24
	Females	1839	31	16	13

P values calculated using ANOVA after controlling for age in parentheses

Serum levels of total carotenes calculated as the sum of *α*-carotene, *β*-carotene, and lycopene levels.

Each serum level of carotenoids was transformed logarithmically.

* : The inhabitants who did not die of cardiovascular disease during follow-up

† : The inhabitants who died of each disease during follow-up

### Hazard Ratios for Serum Carotenoid Values and Cardiovascular Disease Mortality

Based on model 1 (Table 3), high serum values of *α*-carotene, *β*-carotene, lycopene, and total carotene were found to be associated with significantly low hazard ratios (below 1.0) for cardiovascular disease mortality. Those were also related with significant or marginally significant low hazard ratios for heart disease mortality. Using model 2, the hazard ratios for cardiovascular disease mortality also showed significantly low trends for serum values of *α*-carotene, *β*-carotene, and total carotene, and marginally significantly low trends for those of lycopene. However, the hazard ratios for high serum values of carotenoids for mortality from heart disease showed no significantly low trends, except for the serum *α*-carotene value. The significantly low hazard ratios for stroke mortality appeared among subjects with high serum values of *α*-carotene, *β*-carotene, and total carotene (models 1 and 2). In contrast, the hazard ratio for stroke mortality tended to be low among subjects with high serum lycopene values, but it was not a statistically significant risk.

**Table 3.** Hazard ratios (HRs) of serum carotenoid values for cardiovascular disease mortality.

		No		Model 1		Model 2	
		
		Subjects	deaths	HR (95% CI)	p value	HR (95% CI)	p value
Cardiovascular disease	*α*-carotene	3,059	80	0.52 (0.37- 0.74)	0.000	0.55 (0.38- 0.79)	0.001
	*β*-carotene	3,059	80	0.61 (0.45- 0.82)	0.015	0.64 (0.47-0.89)	0.007
	Lycopene	3,059	80	0.73 (0.55-0.97)	0.032	0.77 (0.57-1.04)	0.088
	Total carotene	3,059	80	0.54 (0.38-0.77)	0.001	0.57 (0.40-0.83)	0.003

Heart disease	*α*-carotene	3,059	40	0.45 (0.27-0.73)	0.002	0.51 (0.30-0.86)	0.012
	*β*-carotene	3,059	40	0.70 (0.45-1.09)	0.112	0.84 (0.53-1.34)	0.465
	Lycopene	3,059	40	0.68 (0.45-1.03)	0.069	0.74 (0.48-1.13)	0.163
	Total carotene	3,059	40	0.60 (0.37-0.99)	0.049	0.72 (0.41-1.23)	0.228

Stroke	*α*-carotene	3,059	37	0.56 (0.34-0.94)	0.027	0.52 (0.31-0.88)	0.016
	*β*-carotene	3,059	37	0.53 (0.34-0.82)	0.005	0.48 (0.31-0.76)	0.002
	Lycopene	3,059	37	0.76 (0.50-1.17)	0.210	0.78 (0.51-1.19)	0.253
	Total carotene	3,059	37	0.48 (0.29-0.80)	0.005	0.45 (0.28-0.76)	0.003

Hazard ratios per each logarithmically transformed value of serum carotenoid levels (*μ*mol/L) were calculated using Cox proportional hazard model adjusting for sex, age, smoking status (Model 1), and sex, age, smoking status, alcohol consumption, body mass index, systolic blood pressure, serum total cholesterol levels, and serum values of triglyceride and alanine transaminase activity (Model 2).

Serum levels of carotenoids, triglyceride, and alanine transaminase activity were transformed logarithmically.

Serum levels of total carotenes were calculated as sum of *α*-carotene, *β*-carotene, and lycopene levels.

High serum values of carotenoids were found to have a significant or marginally significant association with low risk for cardiovascular disease mortality (including heart disease and/or stroke), except for the association between high serum lycopene value and stroke mortality, which did not reach statistical significance.

## DISCUSSION

The geographic area studied was located in a rural part of southern Hokkaido, Japan, where a comprehensive health check-up programs, administered by the town office in collaboration with several universities and research institutes, has been offered to inhabitants older than 39 years of age every August from 1986 to the present.^23^^,^^24^ The residents of this area are mainly engaged in dairy farming, commercial fishing, and commerce. The serum carotenoid levels of this cohort were roughly similar to those found in other populations.^26^^-^^28^ For the assay method used in this study, the serum carotenoid determination had a less than 15% coefficient of variation for day to day variation^26^ and individual variations in serum carotenoid levels among the study population tended to be small over 3 years.^29^ Serum levels of *α*-carotene, *β*-carotenes, and lycopene in this population were lower among current smokers and regular alcohol drinkers, which was a finding that has also been reported previously.^23^^,^^30^ Serum levels of total cholesterol and serum enzyme activities reflect liver function and closely correlated with serum levels of carotenoids such as *α*- and *β*-carotenes because almost all serum carotenoids are carried by lipoprotein in the blood and metabolized in the liver.^31^^,^^32^ Because this was not a large-scale population study, we used Cox proportional hazard model with both males and females to estimate the cardiovascular disease mortality risk associated with serum carotenoid values, controlling for sex, age, smoking status, and serum levels of lipids and ALT activity.

Some observational epidemiologic studies have shown that high serum carotenoid levels are associated with a reduced coronary heart disease risk^13^ and that high baseline serum levels of carotenoids such as *α*-carotene and *β*-carotene are inversely and significantly associated with stroke.^18^^,^^33^ In contrast, it has been shown that low serum lycopene levels are associated with an increased risk of atherosclerotic vascular events^17^ and coronary heart disease.^16^^-^^18^

The present results initially obtained by an observational, Japanese population-based, epidemiologic follow-up study showed that high serum values of carotenoids, especially *α*-carotene and *β*-carotene, are significantly and inversely associated with the risk for mortality from cardiovascular disease including stroke, and are in agreement with the results of oversea studies discussed above. High serum lycopene values are not always significantly and inversely related with stroke mortality risk, although they have a marginally significant association with a low cardiovascular disease mortality risk.

Recently, it has been shown that oxidative stress, induced by reactive oxygen species, plays an important role in cardiovascular disease risk through the oxidation of circulating low-density lipoproteins that are key players in the pathogenesis of atherosclerosis and cardiovascular disease.^34^^-^^38^ Inflammation markers, such as highly sensitive C-reactive protein (CRP) and white blood cell counts, have been shown to be significantly higher in patients with an acute ischemic stroke than in controls.^33^ It is well known that carotenoids, such as *α*-carotene, *β*-carotene, and lycopene, have antioxidant activity, and that they protect against oxidative stress such as damage to cell membranes caused by activated oxygen species and free radicals.^20^^-^^22^^,^^36^^,^^38^^,^^39^ Most carotenoids possess bioactivities related to mediating an anti-inflammatory response, which reduces cardiovascular disease risk.^40^

There have been reports indicating that higher serum levels of *α*-carotene, *β*-carotene, and lycopene obtained through a high intake of fruit and vegetables are associated with a lower risk of cardiovascular disease and stroke. In fact, a high intake of such foods can increase serum levels of *α*-carotene and *β*-carotene by about a few tenths of a percent, and bio-factors such as other carotenoids and vitamins (including vitamins B and C) appear to play a role in the prevention of early cardiovascular disease.^10^^-^^12^ Serum levels of carotenoids have been shown to reflect a high intake of colored vegetables; the correlation coefficient between serum levels of carotenoids and the intake frequencies of vegetables such as spinach, carrots, and tomatoes is from 0.20 to 0.30 for *α*-carotene, from 0.21 to 0.33 for *β*-carotene, and from 0.09 to 0.23 for lycopene, respectively. We also found that serum CRP levels in this population were inversely correlated with serum levels of carotenoids (correlation coefficient: -0.19 for *α*-carotene, -0.22 for *β*-carotene, and -0.14 for lycopene, respectively).

These results are consistent in part with the inverse association found between cardiovascular disease mortality and high serum values of *α*-carotene, *β*-carotene, and lycopene observed in the present study.

Our study showed that high serum values of *α*-carotene had a stronger inverse association with a reduced heart disease mortality risk than high serum values of *β*-carotene. Mean serum values of *β*-carotene proved to be over 4-fold higher than those of *α*-carotene in this population. It has been reported that *β*-carotene, when consumed together with fat, is effectively incorporated into a human body,^42^ and that very high *β*-carotene levels appear to lose their antioxidant effectiveness.^43^^,^^44^ These facts may partially depend on the different hazard ratio trends for heart disease mortality between *α*-carotene and *β*-carotene values.

Although low serum lycopene levels were reportedly associated with a high incidence of stroke,^17^ no significant inverse association was found in this study between serum lycopene values and stroke mortality risk. Moreover, this agrees with previously reported results in the American population which indicated that high serum lycopene levels were not associated with a low cardiovascular disease risk,^45^ and that a high dietary lycopene intake was not strongly associated with cardiovascular disease risk.^41^

It has been reported that tomato juice rich in lycopene exhibits many bioactive functions, including acting as an antioxidant, reducing inflammation, inhibiting cholesterol synthesis, improving immune function,^36^^,^^38^^,^^39^ and being beneficial in the prevention of platelet aggregation and thrombosis.^46^

One report showed that, compared to serum *α*- and *β*-carotenes levels, serum lycopene levels were not as strongly associated with inflammation markers such as highly sensitive CRP levels and the NIH Stroke Scale scores, which indicate the severity of neurologic deficits.^34^ It has also been shown that the severity of neurologic deficits strongly predicts the likelihood of a patient’s recovery after stroke.^47^

These findings may depend, in part, on the different hazard ratio trends for stroke mortality among serum values of *α*- and *β*-carotenes and lycopene. They may also be due in part to the higher serum lycopene values found in female subjects who had died than for those who survived. However, the reason why higher serum lycopene values were found only in the females who had died is not clear. This result warrants further study.

In conclusion, the present results indicate that serum levels of *α*-carotene and *β*-carotene as well as lycopene levels may be particularly promising as biomarkers for predicting cardiovascular disease mortality among inhabitants living in a rural area of Japan.
